# Comparison of Major Nutritional Components in *Polygonatum* Germplasm Resources from Different Origins

**DOI:** 10.3390/foods14213663

**Published:** 2025-10-27

**Authors:** Yihong Li, Mei Lu, Qianqian Yang, Qiaojun Jia

**Affiliations:** 1 College of Life Sciences and Medicine, Zhejiang Sci-Tech University, Hangzhou 310018, China; 201411202021.bnu@vip.163.com (Y.L.); 13862044607@163.com (M.L.); yhl@zstu.edu.cn (Q.Y.); 2 Key Laboratory of Plant Secondary Metabolism and Regulation of Zhejiang Province, Zhejiang Sci-Tech University, Hangzhou 310018, China

**Keywords:** *Polygonatum*, nutritional composition, nutritional quality, germplasm

## Abstract

*Polygonatum* has been widely used in traditional Chinese medicine and as a functional food due to its richness in bioactive compounds and nutritional value. However, comprehensive assessments of the nutritional quality of cultivated *Polygonatum* germplasm resources remain limited. In this study, 40 *Polygonatum* germplasms from ten provinces were analyzed for key nutritional components on a dry-weight basis, including polysaccharides, total dietary fiber, vitamin C, protein, and mineral elements. Multivariate analyses (correlation analysis, PCA, membership function scoring, and hierarchical clustering) were applied to comprehensively rank their nutritional quality. Substantial variation was observed across accessions, indicating high genetic diversity. Among them, *Polygonatum cyrtonema* samples from Huaihua (Hunan), Lu’an (Anhui), Hezhou (Guangxi), and Lishui (Zhejiang), as well as *Polygonatum kingianum* from Baoshan (Yunnan), exhibited the highest overall nutritional quality, with polysaccharide content exceeding 20%, and the total dietary fiber and total ash contents greater than 24% and 3%, respectively. This study indicates that the substantial variation observed among different *Polygonatum* germplasm resources increases the likelihood of identifying accessions with high levels of nutritional components, thereby providing a valuable reference for the future industrial development and utilization of *Polygonatum* as both a medicinal and edible plant resource.

## 1. Introduction

*Polygonatum* Mill comprises perennial herbaceous species that are widely distributed across China and have been utilized for both medicinal and nutritional purposes for over 2000 years [1]. *Polygonatum sibiricum* Redoute, *Polygonatum cyrtonema* Hua and *Polygonatum kingianum* Collett & Hemsl are officially listed in the Chinese Pharmacopoeia (2020 edition) [2], and are mainly distributed in the middle and lower reaches of the Yangtze River, such as Guizhou, Zhejiang, Hunan, Hubei, and Sichuan provinces [3,4]. *Polygonatum* is planted under forest conditions, without using additional arable land [5].

The medicinal part of *Polygonatum* is its rhizome, as is the case for *Gastrodia elata* Blume [6], *Pinellia ternate* (Thunb.) Ten. ex Breitenb [7], *Salvia miltiorrhiza* Bunge [8] and *Panax notoginseng* (Burkill) F. H. Chen ex C. H. Chow [9]. *Polygonatum* has long been used in traditional Chinese medicine to tonify Qi, moisten the lung, and strengthen the kidney and spleen [10]. Studies have shown that *Polygonatum* is rich in various compounds, including polysaccharides, flavonoids, saponins, amino acids, and polyphenols, and exhibits multiple functions such as antioxidant, anti-fatigue, hypoglycemic, anti-aging, and immunity enhancement [11,12,13,14,15,16,17]. Current research on the nutritional components of *Polygonatum* has mainly focused on polysaccharides. *Polygonatum* was used as a substitute for grain during times of famine, suggesting that it contains a high level of dietary fiber [18]. *Polygonatum* is also rich in calcium, iron, and amino acids; however, their specific contents in *Polygonatum* remain unclear [19,20].

In addition to its medicinal use, *Polygonatum* is widely recognized as a functional food resource. The mildly sweet taste and chewy texture of processed *Polygonatum* rhizomes make them a suitable ingredient in various functional snacks and baked goods. Modern food processing technologies have further expanded its applications. Various *Polygonatum*-based health products, such as *Polygonatum* wine, tea, yogurt, health beverages, nutritional powders, biscuits, and paste, are available on the market, providing considerable economic benefits to farmers [21,22,23]. Some of the *Polygonatum*-infused products have emerged in convenient formats for daily health supplementation, especially for the elderly population.

As a plant resource with both medicinal and edible properties, *Polygonatum* has attracted widespread interest in recent years. Currently, the annual demand for *Polygonatum* in China is estimated to be between 3500 and 4000 tons [10]. After four years of cultivation, *Polygonatum* can yield approximately 1500 kg of dried rhizomes per hectare [11], with a processed market price of USD 10.5 /kg, resulting in an annual income of approximately USD 15,750 per hectare [5].

The increasing demand for *Polygonatum* has led to the overharvesting of wild resources, resulting in their progressive depletion. Artificial cultivation has been proposed as a viable strategy to meet market demand [24]. However, the accumulation of bioactive components in *Polygonatum* is significantly influenced by origin, species, and cultivation conditions [25,26]. Few studies have systematically assessed the nutritional quality of cultivated *Polygonatum* germplasm resources.

To address this gap, we systematically evaluated the variation in nutritional composition among 40 cultivated *Polygonatum* germplasm resources collected from diverse regions, including Sichuan, Jiangxi, Guangxi, Anhui, Fujian, Zhejiang, Hunan, Shanxi, Yunnan, and Liaoning. Based on the comprehensive nutritional analysis conducted by Lu, which investigated the general nutritional composition, carbohydrate and dietary fiber contents, and the mineral, vitamin, and amino acid profiles of *Polygonatum* germplasms, ten nutritional components were screened as potential indicators for further evaluation of nutritional quality [10]. In the present study, these ten components—polysaccharides, total dietary fiber (TDF), pectin, resistant starch, protein, fructose, vitamin C, iron (Fe), calcium (Ca), and total ash—were selected as the target indicators for assessing the nutritional quality of the 40 *Polygonatum* germplasm resources.

The nutritional profiles of these germplasms were further analyzed using correlation analysis (CA), principal component analysis (PCA), membership function analysis, and cluster analysis. This integrated approach provides a scientific basis for the future development and utilization of *Polygonatum*.

## 2. Materials and Methods

### 2.1. Plant Materials

In total, 40 *Polygonatum* germplasms were collected from Sichuan, Jiangxi, Guangxi, Hunan, Anhui, Zhejiang, Fujian, Yunnan, Shanxi and Liaoning provinces (Table S1), and were cultivated in Yuhang district, Hangzhou city, Zhejiang province, China. All germplasms were cultivated under identical agronomic conditions in Yuhang prior to analysis. The plants were harvested after three years. All samples were authenticated by Prof. Zongsuo Liang (Zhejiang Sci-Tech University), assigned voucher specimen numbers, and deposited at the Key Laboratory of Plant Secondary Metabolism and Regulation of Zhejiang Province. For each germplasm, rhizomes from three individual plants were independently processed to generate three biological replicates. Each biological replicate was measured in triplicate during analytical assays. The rhizomes of plants were washed and sliced, with three biological replicates. All samples were dried in a forced-air oven at 55 °C until a constant weight was achieved, and subsequently ground into a fine powder (60 mesh). The powder was packaged in sealed plastic bags and stored in dry glassware at room temperature.

### 2.2. Reagents

The following reagents were purchased: fructose, glucose, sucrose, kestose, nystose, fructofuranosylnystose, potassium ferrocyanide trihydrate, pure distilled water, ethanol (Sangon Biotech, Shanghai, China; A422957, A430191, A361851, A350827, A350828, A418962, A600768, B548405, A375262), acetonitrile and triethylamine (Merck, Darmstadt, Germany; 10030, 808352), α-amylase, protease, glucoamylase, and hydrogen peroxide (Sigma-Aldrich, St. Louis, MI, USA; A6380, P5380, A7420, Z100970).

### 2.3. Sweetness Value Analysis

The three standards of glucose, fructose and sucrose were prepared into mixed standard solutions with different concentration gradients in the range of 0.5–6 mg/mL. An amount of 0.050 g of prepared powder from the 40 samples was weighed and extracted with 1.8 mL of water as the internal reference for 1 h in an ultrasonic bath at 4 °C. After centrifuging at 9500 rpm/min at 4 °C for 10 min, the supernatant was filtered with a 0.45 µm microporous membrane and then used for HPLC analysis.

An HPLC system (Waters, Milford, CT, USA; e2695) coupled with PCA was used for the analysis of glucose, fructose, and sucrose. The samples were first separated using an XBridge Amide column (250 mm × 4.6 mm, particle size 5 μm). The column temperature was kept constant at 35 °C, and the flow rate was 1 mL/min with an injection volume of 15.0 µL. The mobile phases for gradient elution consisted of water (solvent A) and 0.2% (*v*/*v*) triethylamine/acetonitrile (solvent B), with an approximate pH of 7.5–8.0. The elution gradients were 15–25% A over 0–10 min, 25% A over 10–50 min, and 25–75% A over 50–55 min. Calibration curves for each sugar standard were constructed within the range of 0.5–6 mg/mL, with correlation coefficients (R^2^ > 0.999). Method repeatability was assessed by triplicate injections of quality control samples, yielding RSD values below 2.3%.

The sweetness value was calculated as 1.75 times the fructose content, 0.7 times the glucose content, and the sucrose content added together [27].

### 2.4. Determination of Polysaccharide Content, Kestose and Nystose

An amount of 1.0 g of *Polygonatum* powder was weighed and extracted with water at a solid-to-liquid ratio of 1:20. The mixture was incubated in a water bath for 2.5 h, and then centrifuged to collect the supernatant. Anhydrous ethanol was added to the supernatant to adjust the ethanol concentration to 80%, and the solution was kept at low temperature overnight for ethanol precipitation. After centrifugation, the supernatant was discarded, and the precipitate was dissolved in water. The polysaccharide content was then determined by sulfuric acid–anthrone method [28,29].

The determination of the content of kestose and nystose followed the same procedure as that in Section 2.3.

### 2.5. Determination of Fiber Composition, Protein and Ash Contents

The resistant starch (RS) content was determined by the enzyme digestion method [30]. Briefly, 0.1 g prepared powder of 40 samples was weighed and mixed with 4 mL of an α-tryptic amylase suspension. The mixture was shaken at 37 °C for 16 h. Then, 4 mL of anhydrous ethanol and 8 mL of 50% ethanol were added. After removing the supernatant, the precipitate was treated with 2 mL of 2 mol/L KOH, 8 mL of 1.2 mol/L sodium acetate buffer (pH 3.8), and 1 mL of amyl glucosidase (AMG) at 50 °C for 30 min. The glucose content was determined using the 3,5-dinitrosalicylic acid (DNS) method. The resistant starch content of the sample was calculated by multiplying the measured glucose content by 0.9.

The pectin content was analyzed using a spectrophotometer. An amount of 0.1 g of prepared powder from the 40 samples was weighed and mixed with anhydrous ethanol at 85 °C for 10 min. The precipitate was then mixed with 10 mL of distilled water and 0.5 mL of 40 g/L NaOH and agitated for 15 min. An amount of 1 mL of supernatant was mixed with 0.25 mL of carbazole ethanol and 5 mL of concentrated sulfuric acid, and then the mixture was placed at 85 °C for 20 min. After quick cooling, the absorbance was measured at 525 nm [31].

After desugarization, drying, and sieving, duplicate specimens were enzymatically digested using heat-stable α-amylase, protease, and glucoamylase, followed by precipitation with 95% ethanol and filtration. The residue was sequentially washed with 78% ethanol, 95% ethanol, and acetone, and then dried and weighed. The protein and ash contents of the residue were subsequently determined, and the dietary fiber content was calculated according to the corresponding formula [32].

$$Total\;dietary\;fiber(\%)=\frac{{\bar{m}}_{R}-m_{P}-m_{A}-m_{B}}{\bar{m}\times \frac{m_{C}}{m_{D}}}\times 100$$

where ${\bar{m}}_{R}$ = mean weight of double sample residues, $m_{P}\;$ = weight of protein in sample residue, $m_{A}$ = weight of ash in sample residue, $m_{B}$ = weight of empty, $\bar{m}$ = mean weight of double samples, $m_{C}$ = weight of sample before desugaring step, and $m_{D}$ = weight of sample after desugaring step.

### 2.6. Determination of Other Nutritional Components

Vitamin C concentration was determined spectrophotometrically with fast blue B salt (FBSB) [33]. Vitamin C reacts with FBSB to generate a yellow oxalohydrazide-2-hydroxybutyryl lactone derivative. The linearity range is 0–20 μg/mL, and the calculated limit of detection (LOD) and limit of quantification (LOQ) are 0.12 μg/mL and 0.40 μg/mL, respectively, with R^2^ > 0.999. There is no interference from coexisting sugars, organic acids, proteins, amino acids, etc. An amount of 0.1 g of prepared powder from the 40 samples was weighed and extracted with 0.5 mL of 2 mol/L acetic acid, 0.2 mL of 0.25 mol/L EDTA and 4.3 mL of anhydrous ethanol as the internal reference for 15 min in an ultrasonic bath at 4 °C. Then, 5 mL water was added, and the mixture was ultra-sounded for 15 min. An amount of 600 μL of the supernatant was mixed with 200 μL of water, 50 μL of 2 mol/L acetic acid, 100 μL of 0.25 mol/L EDTA, and 50 μL of 0.6% FBSB. After centrifuging at 8500 rpm/min at 4 °C for 5 min, the absorbance of the supernatant was measured at 420 nm.

The content of Ca and Fe of each sample was determined by atomic absorption spectroscopy. An amount of 0.2 g of prepared powder from the 40 samples was weighed and mixed with nitric acid (10 mL) and hydrogen peroxide (2 mL) in a polytetrafluoroethylene sample cup. A digestion tank was assembled and placed in a microwave digestion system (Shanghai Metash Instruments Co., Ltd., Shanghai, China; MWD630) for sample digestion. After cooling to 60 °C, the digestion tanks were removed for acid removal. The digested sample was then transferred into a 50 mL volumetric flask and diluted to a given volume with distilled water. The sample solution was filtered with a 0.45 µm microporous membrane and then used for analysis [34]. Calibration was conducted using an external standard method with five concentration levels (1, 2, 5, 10, and 20 mg/L for Fe; 5, 10, 20, 40, and 80 mg/L for Ca). Quality control was ensured by including procedural blanks and spike recovery tests, which yielded recoveries of 94.5–102.3% for Fe and 92.8–101.6% for Ca. The LODs were 0.02 mg/kg (Fe) and 0.05 mg/kg (Ca), and the LOQs were 0.07 mg/kg and 0.15 mg/kg, respectively.

### 2.7. Statistical Analysis

All compositional data, including those for polysaccharides, total dietary fiber, resistant starch, sugars, protein, vitamin C, Fe, and Ca, were determined on a dry-weight (DW) basis. For each germplasm, rhizomes from three individual plants were collected as biological replicates. Each biochemical assay was performed in triplicate for each biological replicate. The means ± SD presented reflect biological variation across the three independent samples. Data analysis was performed using GraphPad Prism 9 and Origin 2021. Significance testing was conducted using one-way ANOVA based on biological replicates.

## 3. Results

### 3.1. Sweetness Evaluation and Polysaccharide Content of Polygonatum Germplasms

Fructose, glucose, and sucrose are the main soluble sugars contributing to the sweetness of foods. To investigate the variation in sweetness among 40 *Polygonatum* germplasm resources, we quantified the contents of fructose, glucose, and sucrose. The results showed significant differences in sugar content across the different *Polygonatum* germplasms.

Fructose contents exhibited the greatest variability, ranging from 0.77% to 9.28%, with an average content of 2.78% and a coefficient of variation (CV) of 73.49%. The highest fructose contents were observed in SXLB (9.28%), GXHZ (7.67%), FJSM04 (6.53%), and HNHH01 (6.13%), while SCNC01, JXYC, GXGL, and ZJJN01 displayed the lowest levels (all around 0.80%). Sucrose contents ranged from 1.44% to 7.98%. HNHH01 (7.98%), HNHH02 (7.92%), and HNHH03 (7.94%) showed the highest sucrose contents, while SCNC01 (1.72%), ZJJN01 (1.44%), and ZJAJ (1.54%) exhibited the lowest sucrose contents. Glucose contents ranged from 0.33% to 2.81%. FJSM04 had the highest glucose content (2.81%), while JXYC, GXGL, AHCZ04, and YNKM02 presented relatively low levels (approximately 0.34%) (Table 1).

**Figure 1:**
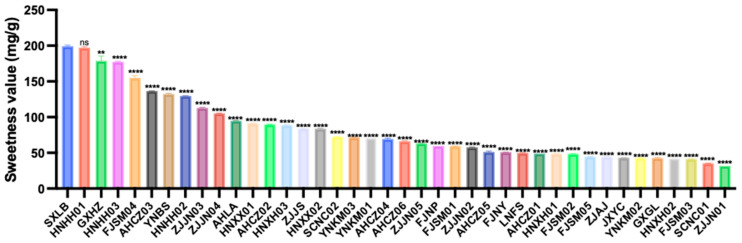
Sweetness values of the 40 *Polygonatum* germplasms. Data are presented as means ± SD (*n* = 3). ns *p* > 0.05, ** *p* < 0.01, **** *p* < 0.0001.

Based on the sweetness values of the soluble sugars, the overall sweetness of the 40 germplasms ranged from 31.83 to 199.12 mg/g, with an average value of 82.91 mg/g and a CV of 56.17%. Among them, SXLB exhibited the highest sweetness value (199.12 mg/g), whereas ZJJN01 had the lowest (31.83 mg/g) (Figure 1).

To evaluate the variation in polysaccharide contents among different *Polygonatum* germplasms, we measured the differences in polysaccharide contents. Among them, HNXH02 exhibited the highest polysaccharide content (27.64%), while HNHH01, HNHH02, AHCZ04, FJNY, and YNKM01 showed relatively low levels, all around 8% (Table 1).

### 3.2. Analysis of Dietary Fiber Content in 40 Polygonatum Germplasms

To investigate the total dietary fiber (TDF) content in the 40 *Polygonatum* germplasm samples, enzymatic-gravimetric assays were performed. The results showed that the TDF content ranged from 14.84% to 25.82%, with an average of 19.44%. AHLA, ZJJN04, FJSM04, AHCZ06, and YNBS exhibited relatively high TDF levels (all over 24%), while ZJJS, ZJAJ, FJSM03, FJNP, YNKM01, and YNKM03 showed comparatively low levels (approximately 15%).

To further characterize the soluble dietary fiber profile in the 40 *Polygonatum* germplasm samples, we quantified the contents of resistant starch, kestose, nystose, and pectin. Resistant starch was the most abundant soluble dietary fiber among the measured components. HNXX01 had the highest resistant starch content (2.01%), while ZJAJ, FJSM02, and FJSM05 exhibited comparatively low levels. The contents of kestose in different samples were significantly different with a CV of 71.39%. HNHH01 had the highest kestose content (1.38%), whereas SCNC01 had the lowest (0.18%). YNKM03 showed the highest nystose content (0.74%), while AHCZ06, HNXH01, FJNY, and SCNC01 displayed relatively low levels. Pectin content was higher in ZJJN01, ZJAJ, FJSM02, FJSM04, and FJSM05, whereas lower levels were observed in JXYC, GXGL, and HNXH02 (Table 2).

### 3.3. Analysis of Other Nutritional Components and Total Major Nutrient Contents in Polygonatum Germplasms

To investigate the variation in other nutritional components among the 40 *Polygonatum* samples, we quantified the contents of protein, vitamin C, total ash, Fe, and Ca. The protein content in the 40 *Polygonatum* samples ranged from 3.06% to 8.97%. Higher protein levels were observed in GXHZ, HNXH02, HNXX02, AHLA, and AHCZ06 (all over 6.80%), while those in AHCZ05, ZJAJ, and YNKM0103 were relatively low (all below 4.20%). Vitamin C content ranged from 67.79 to 166.53 mg/100 g. Among the samples, HNHH01 had the highest vitamin C content (166.53 mg/100 g), while AHCZ05, ZJJN02, and YNKM0103 showed the lowest levels (approximately 70 mg/100 g). The total ash content ranged from 1.18% to 3.80%, and ZJJN03, ZJJN04, ZJJS, and FJSM04 showed relatively high levels, while AHCZ05 had the lowest ash content (1.18%) (Table 3).

The Fe content among the 40 *Polygonatum* samples ranged from 9.98 to 44.40 mg/100 g, with a CV of 34%, indicating significant differences in Fe levels among the different *Polygonatum* germplasms. HNHH01 exhibited the highest Fe content (44.40 mg/100 g), whereas HNXH01, AHCZ04, AHCZ05, and ZJJN01 showed relatively low Fe levels. The Ca content ranged from 160.66 to 565.32 mg/100 g. FJSM04 had the highest Ca content (565.32 mg/100 g), while SCNC02, ZJAJ, FJSM01, FJSM03, and LNFS exhibited comparatively low Ca contents (Table 3).

**Figure 2:**
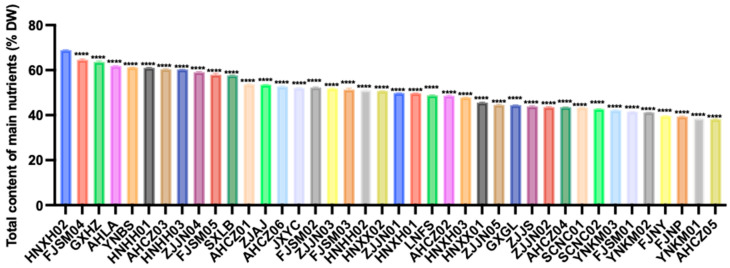
Total nutrient content of the 40 *Polygonatum* germplasm. Data are presented as means ± SD (*n* = 3). **** *p* < 0.0001.

Based on a weighted composite score of the nutritional indicators, the 40 *Polygonatum* germplasm samples were ranked by total nutrient content (Figure 2). The top ten were HNXH02, FJSM04, GXHZ, AHLA, YNBS, HNHH01, AHCZ03, HNHH03, ZJJN04, and FJSM05. In contrast, FJNY, FJNP, YNKM01, and AHCZ05 were among the samples with relatively low overall nutrient levels.

### 3.4. Correlation Analysis Among Nutritional Components in Polygonatum Germplasms

To explore the relationships among different nutritional qualities in *Polygonatum* germplasm samples, we conducted a correlation analysis. The results revealed complex correlations among various nutritional components, especially among polysaccharides, total ash, total dietary fiber and kestose (Table S2).

Correlation analysis showed that polysaccharide content was highly significantly positively correlated with pectin, and significantly negatively correlated with resistant starch, sucrose, kestose, and nystose. The total ash exhibited highly significant positive correlations with fructose, glucose, Ca, total dietary fiber, protein, kestose, and Fe. TDF was highly significantly positively correlated with protein and significantly positively correlated with vitamin C, sucrose and Ca. Kestose was significantly positively correlated with pectin, glucose, sucrose, nystose, and Ca, and showed a highly significant positive correlation with nystose. Pectin was highly significantly positively correlated with glucose and sucrose. Vitamin C was highly significantly positively correlated with sucrose, and significantly positively correlated with protein, fructose, Fe, and total dietary fiber. No significant correlations were observed among the other nutritional components (Table S2).

### 3.5. Principal Component Analysis and Membership Function Evaluation in Polygonatum Germplasm

To gain a more comprehensive understanding of the variation among 40 *Polygonatum* germplasm samples, principal component analysis (PCA) was performed on the major nutritional components. It is generally accepted that principal components with a cumulative contribution of variance exceeding 80% are representative of the original dataset. Therefore, the first six principal components, which together accounted for 83.83% of the total variance, were selected to represent the majority of the initial information contained in the nutritional profiles of the 40 *Polygonatum* germplasm samples.

Dietary fiber, fructose, glucose, and ash were shown to be the most influential components in the positive region of principal component 1 (PC1). Resistant starch had a greater positive influence on PC2, whereas polysaccharides and pectin exhibited a strong negative influence on PC2. PC3 was primarily dominated by protein, while kestose and nystose had major negative contributions. PC4 was mainly characterized by sucrose, Fe, and Ca. PC5 was mainly dominated by protein and Fe, and PC6 primarily reflected the variation in total ash content (Table 4).

**Figure 3:**
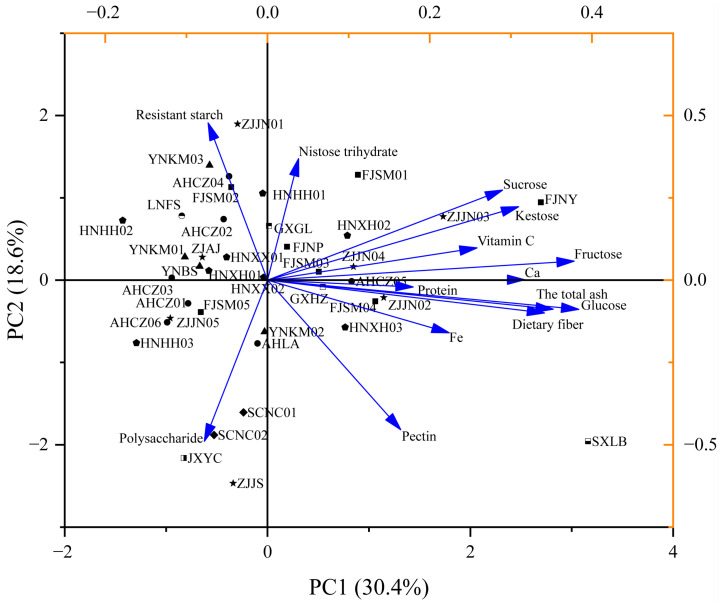
Loading and score plot of PCA for the nutrients of the 40 *Polygonatum* germplasm resources. PC1 (30.4%) and PC2 (18.6%) represent the percentage of explained variance.

The 40 *Polygonatum* germplasm samples from different geographical origins were relatively scattered in the PCA plot (Figure 3), and principal components 1 and 2 could be used to distinguish most of the germplasm lines. SXLB was located in the lower-right quadrant of the plot, characterized by high levels of glucose, ash, and dietary fiber. FJNY appeared in the upper-right quadrant, exhibiting relatively low polysaccharide content but high levels of kestose and sucrose. ZJJN01 was located in the upper left quadrant, showing high levels of resistant starch but low levels of other major nutrients. JXYC, ZJJS, SCNC01, and SCNC02 clustered in the lower-left quadrant, sharing similar nutritional profiles marked by higher polysaccharide content and relatively low concentrations of other major nutrients.

To further analyze the nutritional quality of the 40 *Polygonatum* germplasm samples, a comprehensive nutritional score (Y) was calculated and ranked using the following formula:

Y = Y_1_ × 30.43% + Y_2_ × 18.61% + Y_3_ × 14.33% + Y_4_ × 8.88% + Y_5_ × 6.47% + Y_6_ × 5.10% (Table 5). HNHH01 had the highest nutritional quality score, characterized by high levels of vitamin C, fructose, glucose, sucrose, kestose, Fe, and total dietary fiber. HNHH03, HNHH02, GXHZ, HNXX02, AHLA, HNXH02, YNBS, AHCZ04 and ZJJN04 had higher nutritional quality scores, and they generally exhibited relatively high contents of ash, protein, fructose, sucrose, Ca and total dietary fiber.

The membership function method was applied for comprehensive nutritional quality evaluation because it allows for the normalization of multiple indicators with different units into comparable dimensionless scores. This approach has been widely used in germplasm quality assessment and crop cultivar ranking, where multiple nutritional or agronomic traits must be integrated into a single index [35]. To validate and supplement the results of the nutritional quality scoring method, a membership function analysis was also conducted (Table S3). Based on this approach, the top ten samples in terms of nutritional quality were HNHH01, FJSM04, HNHH03, AHLA, YNBS, GXHZ, HNHH02, SXLB, ZJJN04, and ZJJN03. Notably, HNHH03, HNHH02, GXHZ, AHLA, YNBS, and ZJJN04 consistently ranked high in nutritional quality under principal component analysis and membership function analysis, indicating superior nutritional performance.

### 3.6. Cluster Analysis of Polygonatum Germplasm

**Figure 4:**
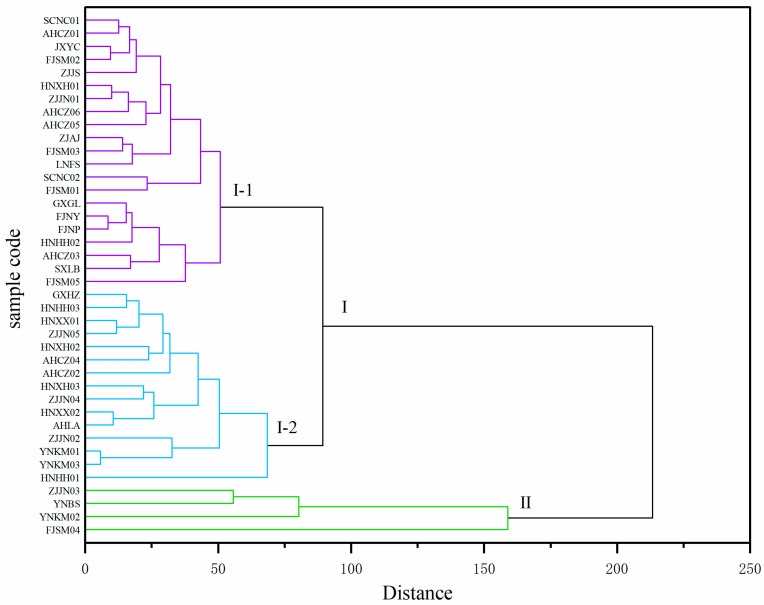
Cluster analysis of the 40 *Polygonatum* germplasm resources (Euclidean distance, Ward’s method). The dendrogram was cut at distance 200 to define two major clusters (I and II), with Cluster I further subdivided into two subgroups (I-1 and I-2).

To identify the 40 *Polygonatum* germplasm samples with specific dominant nutritional traits, cluster analysis was conducted by using 14 nutritional quality indicators, with Euclidean distance and Ward’s minimum variance method. When the Euclidean distance was 200, the germplasm samples were divided into two major clusters (Figure 4). Cluster I included 36 samples, and Cluster II contained ZJJN03, FJSM04, YNKM02, and YNBS, which were characterized by relatively high levels of dietary fiber and Ca content. When the Euclidean distance threshold was reduced to 80, Cluster I could be further divided into two subgroups, Cluster I-1 (21 samples) and Cluster I-2 (15 samples), the latter of which exhibited higher contents of fructose, sucrose, kestose, nystose, vitamin C, Ca, and dietary fiber compared to those in Cluster I-1.

## 4. Discussion

Polysaccharides possess a wide range of biological activities, including hypoglycemic, hypolipidemic, anti-tumor, anti-inflammatory, anti-fatigue, and anti-aging effects, as well as immune enhancement and the regulation of intestinal microbial communities [12,36,37,38]. Polysaccharide content is considered as an important indicator for evaluating the nutritional quality of *Polygonatum* [39]. The polysaccharide content of the 40 Polygonatum germplasm samples ranged from 7.73% to 27.64%, not only exceeding the minimum requirement of 7% stipulated in the Chinese Pharmacopoeia (2020 edition), but also being markedly higher than the levels typically reported for other medicinal plants such as *Codonopsis pilosula* (Franch.) Nannf, *Dendrobium officinale* Kimura & Migo, and *Ophiopogon japonicus* (L. f.) Ker Gawl (5–15%) [2,40,41,42]. The polysaccharide levels measured in *Polygonatum cyrtonema* were generally higher than those reported for *Polygonatum kingianum* (7.84–15.12%) and *Polygonatum sibiricum* (9.56–16.26%), consistent with the findings of Jiao [43], and were also higher than those in *Polygonatum odoratum* (Mill.) Druce [44]. In addition, the range observed in this study was higher than that of the polysaccharide content reported by Jiao for 33 wild *Polygonatum sibiricum* samples (2.23–14.09%) [43], and was greater than the polysaccharide content range of 18 wild *Polygonatum sibiricum* determined by Wang (9.56–17.68%) [45], suggesting that artificial cultivation is conducive to polysaccharide accumulation in *Polygonatum*.

We found that the CV for fructose, glucose, sucrose, kestose, nystose, and pectin contents were relatively high, at 73.49%, 68.32%, 52.87%, 71.39%, 46.95%, and 60.18%, respectively, indicating substantial genetic variation among *Polygonatum* germplasms with respect to these nutritional components. Similar patterns have been reported in *Codonopsis* from different sources, where significant differences in the levels of fructose, glucose, sucrose, kestose, and nystose were observed [46], suggesting that these small-molecule sugars can serve as effective markers for distinguishing germplasm in medicinal plants.

Sweetness is an important factor influencing consumers’ food choices [47]. In the present study, we found that the fructose and sucrose contents across different *Polygonatum* germplasm samples were relatively similar and generally higher than that of glucose, indicating that fructose and sucrose are the main sources of sweetness in *Polygonatum*. Furthermore, the sweetness values observed in this study (ranging from 31.83 to 199.12 mg/g) were greater than those reported for persimmon fruits (3.04–44.25 mg/g) [47] and fresh sweet potato (25.41–86.80 mg/g) [48], but lower than those of jujube fruits (119.43–369.75 mg/g) [49]. These results indicate that *Polygonatum* germplasm resources hold great potential for selective breeding aimed at improving sweetness, flavor, and carbohydrate-based functional properties. As the sweetness coefficients applied in this study were adopted from literature values rather than obtained through sensory validation, the calculated sweetness should be regarded as a theoretical estimate. Future work incorporating consumer-based sensory assessment would help refine this metric for practical applications.

TDF can prevent and alleviate a variety of diseases, including type 2 diabetes, cardiovascular disease, and colon cancer [50]. The TDF content of the 40 *Polygonatum* samples was greater than 6%, which meets the threshold for high-dietary-fiber foods as defined by the Chinese National Standard GB 28050-2011 [51]. Moreover, among all measured nutritional indicators, TDF and resistant starch showed the lowest CV, at 16.66% and 13.48%, respectively, indicating that the contents of these two components were relatively stable across different *Polygonatum* germplasm sources. The TDF content in the 40 *Polygonatum* germplasms was higher than that in root vegetables such as potato, cassava, beetroot and radish [52,53,54,55].

The protein content of the 40 *Polygonatum* samples ranged from 3.06% to 8.97%, greater than the range reported by Li for 35 *Polygonatum* samples from different origins (3.23–4.97%) [56]. The vitamin C content (67.79–166.53 mg/100 g) was comparable to that of some kiwifruit varieties [57], indicating that *Polygonatum* is rich in vitamin C. The Fe content range of the 40 samples was slightly higher than that reported for *Polygonatum odoratum* as measured by Liu (4.25~36.28 mg/100 g) (Liu et al., 2022), and the Ca content range is also slightly higher than the result measured by Huang (122.54~469.03 mg/100 g) [58]. Mineral analyses revealed abundant Fe and Ca, comparable to or exceeding levels reported for *Polygonatum odoratum* [55], further highlighting the nutritional and pharmacological potential of cultivated *Polygonatum*. These results suggest that the nutrient contents of *Polygonatum* from different sources after artificial cultivation are generally higher than those reported for wild *Polygonatum*.

The correlation analysis revealed complex interrelationships among the ten key nutritional components of *Polygonatum*, suggesting coordinated accumulation and potential metabolic linkage among certain nutrients. For instance, the significant positive correlation between polysaccharides and pectin, coupled with their simultaneous negative correlation with resistant starch, is consistent with Lu’s findings [10], possibly reflecting divergent carbon partitioning pathways during storage and polysaccharide biosynthesis [59]. Studies have shown that when the accumulation of sugars in chestnuts decreases, the accumulation of starch reaches its peak [60]. The negative associations of polysaccharides with sucrose, kestose, and nystose further support this hypothesis, indicating a metabolic trade-off between different carbohydrate types. This pattern was further supported by the PCA results, where polysaccharides exhibited a strong negative loading on PC2 (−0.490), while resistant starch showed a marked positive loading, suggesting that these carbohydrate-related nutrients may undergo coordinated but opposing accumulation trends. Such antagonistic relationships likely reflect the preferential allocation of carbon resources under specific genetic or environmental contexts.

It should be noted that the ‘top-ranked’ germplasms based on total nutrient content differ slightly from those selected by PCA or membership function scoring. This discrepancy arises because simple cumulative nutrient content gives equal weight to each component, whereas PCA and membership scoring assign weights based on variance contribution and multidimensional performance. Therefore, accessions performing exceptionally well in one nutrient may rank differently when holistic weighting is applied.

In addition, the significant correlations between vitamin C and various nutrients including sucrose, protein, fructose, Fe, and total dietary fiber may reflect its broad involvement in primary metabolism and redox balance, which aligns with Uetaki’s work [61]. These findings show the intricate nutritional interdependencies within the 40 *Polygonatum* germplasms and lay a theoretical foundation for targeted breeding strategies.

Previous reports have demonstrated that cultivated *Polygonatum sibiricum* contains markedly higher levels of multiple bioactive constituents compared with its wild counterparts, with up to 22 phytochemicals showing significant elevation under cultivation conditions [62]. Our analysis reveals substantial variation in nutrient composition among the 40 cultivated germplasms in this study. This intra-cultivar divergence is postulated to be driven primarily by genetic background, as genotypic differences are known to exert strong control over metabolic profiles in *Polygonatum* [63]. Microenvironmental differences may contribute to this divergence, but genetic background is the primary driver of nutritional disparities. Thus, although environmental factors likely play a supportive role, our findings suggest that genetic divergence among germplasms shows a stronger influence on nutrient accumulation. Future breeding and germplasm selection should place greater emphasis on genetic background to achieve stable improvements in *Polygonatum* quality.

In conclusion, this study provides a comprehensive nutritional assessment of 40 *Polygonatum* germplasm resources from different regions of China. The results demonstrate substantial variation in nutrient composition, reflecting both genetic diversity and environmental adaptability. Cultivated *Polygonatum cyrtonema* and *Polygonatum kingianum* exhibited particularly high nutritional quality, characterized by elevated polysaccharide, dietary fiber, and mineral contents. Given their rich nutritional profile, *Polygonatum* species show strong potential for development as functional food ingredients. The polysaccharides and dietary fibers identified in this study could be further explored for health-promoting properties such as glycemic control, antioxidant protection, and gut microbiota modulation.

Future studies should focus on elucidating the molecular mechanisms underlying nutrient biosynthesis and accumulation in *Polygonatum*, and on linking specific compounds to biological activities through metabolomic and pharmacological validation. Although this study is limited by the absence of experimental validation of bioactive properties and incomplete metabolite profiling, which prevented further confirmation of the functional roles of key nutrients, it nevertheless highlights the nutritional value of *Polygonatum* as both a medicinal and edible plant resource and provides valuable data to support its development and industrialization—particularly in the functional food sector.

## Figures and Tables

**Table 1:** The contents of polysaccharide, fructose, glucose and sucrose in 40 *Polygonatum* germplasm resources.

Sample Code	Polysaccharide/% DW	Fructose/% DW	Glucose/% DW	Sucrose/% DW	Sweetness Value/mg/g DW
SCNC01	12.83 ± 0.14 ^i^	0.87 ± 0.01 ^vw^	0.41 ± 0.00 ^pqrs^	1.72 ± 0.02 ^q^	35.35
SCNC02	10.89 ± 0.05 ^kl^	2.14 ± 0.07 ^n^	0.52 ± 0.02 ^hijk^	3.16 ± 0.13 ^de^	72.69
JXYC	20.36 ± 0.20 ^d^	0.85 ± 0.01 ^w^	0.33 ± 0.00 ^t^	2.59 ± 0.01 ^jk^	43.12
GXGL	10.20 ± 0.02 ^mno^	0.77 ± 0.02 ^w^	0.34 ± 0.00 ^t^	2.69 ± 0.04 ^hij^	42.78
GXHZ	15.58 ± 0.12 ^g^	7.67 ± 0.33 ^b^	0.44 ± 0.02 ^noppq^	4.16 ± 0.08 ^b^	178.81
HNXH01	16.52 ± 0.06 ^f^	1.40 ± 0.04 ^rst^	0.41 ± 0.02 ^qrs^	2.13 ± 0.05 ^n^	48.67
HNXH02	27.64 ± 0.15 ^a^	1.11 ± 0.01 ^u^	0.39 ± 0.00 ^rs^	2.05 ± 0.02 ^n^	42.63
HNXH03	10.67 ± 0.13 ^klm^	2.91 ± 0.02 ^k^	0.56 ± 0.01 ^h^	3.38 ± 0.04 ^c^	88.61
HNHH01	8.68 ± 0.11 ^r^	6.13 ± 0.04 ^d^	1.46 ± 0.01 ^b^	7.98 ± 0.06 ^a^	197.27
HNHH02	7.73 ± 0.05 ^t^	2.53 ± 0.02 ^lm^	0.88 ± 0.02 ^e^	7.92 ± 0.07 ^a^	129.63
HNXX01	10.16 ± 0.06 ^mnop^	3.22 ± 0.02 ^j^	0.62 ± 0.01 ^g^	3.05 ± 0.01 ^f^	91.13
HNXX02	9.79 ± 0.29 ^nopq^	3.02 ± 0.04 ^k^	0.56 ± 0.01 ^hi^	2.70 ± 0.01 ^hi^	83.81
AHLA	15.09 ± 0.20 ^g^	3.47 ± 0.02 ^i^	1.20 ± 0.01 ^c^	2.57 ± 0.01 ^k^	94.91
AHCZ01	20.42 ± 0.20 ^d^	1.28 ± 0.01 ^st^	0.38 ± 0.01 ^st^	2.41 ± 0.01 ^m^	49.11
AHCZ02	12.55 ± 0.16 ^i^	2.60 ± 0.01 ^l^	0.47 ± 0.01 ^klmno^	4.11 ± 0.01 ^b^	89.94
AHCZ03	18.26 ± 0.16 ^e^	5.18 ± 0.02 ^f^	0.74 ± 0.01 ^f^	4.06 ± 0.03 ^b^	136.55
AHCZ04	8.36 ± 0.11 ^rs^	1.88 ± 0.05 ^op^	0.36 ± 0.01 st	3.41 ± 0.03 ^c^	69.45
AHCZ05	9.37 ± 0.04 ^q^	1.56 ± 0.03 ^qr^	0.64 ± 0.04 ^g^	1.94 ± 0.05 ^o^	51.18
ZJJN01	20.46 ± 0.27 ^d^	0.82 ± 0.00 ^w^	0.44 ± 0.01 ^noppq^	1.44 ± 0.00 ^s^	31.83
ZJJN02	9.54 ± 0.08 ^q^	2.02 ± 0.02 ^no^	0.45 ± 0.01 ^mnopq^	1.93 ± 0.01 ^o^	57.88
ZJJN03	9.71 ± 0.29 ^opq^	4.47 ± 0.04 ^g^	0.75 ± 0.01 ^f^	2.94 ± 0.03 ^g^	112.85
ZJJN04	14.22 ± 0.21 ^h^	3.85 ± 0.01 ^h^	0.64 ± 0.00 ^g^	3.36 ± 0.02 ^c^	105.36
ZJJN05	11.96 ± 0.20 ^j^	1.67 ± 0.01 ^q^	0.46 ± 0.00 ^lmnop^	3.08 ± 0.01 ^ef^	63.28
ZJJS	11.06 ± 0.16 ^k^	3.19 ± 0.01 ^j^	0.62 ± 0.00 ^g^	2.37 ± 0.02 ^m^	83.92
ZJAJ	25.33 ± 0.25 ^b^	1.46 ± 0.03 ^rs^	0.51 ± 0.02 ^ijk^	1.54 ± 0.02 ^r^	44.47
FJSM01	10.6 ± 0.07 ^klm^	1.84 ± 0.02 ^p^	0.49 ± 0.01 ^jklmn^	2.38 ± 0.01 ^m^	59.43
FJSM02	19.93 ± 0.12 ^d^	1.02 ± 0.01 ^uv^	0.43 ± 0.01 ^opqr^	2.77 ± 0.03 ^h^	48.60
FJSM03	21.92 ± 0.50 ^c^	1.08 ± 0.00 ^u^	0.44 ± 0.01 ^mnopq^	1.93 ± 0.01 ^o^	41.39
FJSM04	16.46 ± 0.20 ^f^	6.53 ± 0.16 ^c^	2.81 ± 0.08 ^a^	2.10 ± 0.04 ^n^	155.01
FJNY	7.84 ± 0.03 ^st^	1.29 ± 0.01 ^st^	0.45 ± 0.01 ^mnopq^	2.53 ± 0.01 ^kl^	50.96
FJNP	11.17 ± 0.22 ^k^	2.17 ± 0.01 ^n^	0.50 ± 0.01 ^jklm^	1.82 ± 0.01 ^pq^	59.68
AHCZ06	10.35 ± 0.11 ^lmn^	2.37 ± 0.02 ^m^	0.51 ± 0.01 ^ijk^	2.13 ± 0.03 ^n^	66.34
YNKM01	7.84 ± 0.16 st	2.47 ± 0.01 ^lm^	0.51 ± 0.01 ^jkl^	2.45 ± 0.02 ^lm^	71.27
YNKM02	9.59 ± 0.12 ^pq^	1.27 ± 0.01 ^t^	0.33 ± 0.01 ^t^	1.85 ± 0.03 ^op^	42.97
YNKM03	10.85 ± 0.26 ^kl^	2.06 ± 0.06 ^n^	0.54 ± 0.02 ^hij^	3.19 ± 0.08 ^d^	71.77
YNBS	15.12 ± 0.18 ^g^	5.86 ± 0.05 ^e^	0.53 ± 0.01 ^hij^	2.61 ± 0.02 ^ijk^	132.42
HNHH03	9.56 ± 0.09 ^q^	5.23 ± 0.03 ^f^	0.92 ± 0.02 ^de^	7.94 ± 0.06 ^a^	177.34
SXLB	10.96 ± 0.16 ^kl^	9.28 ± 0.07 ^a^	0.96 ± 0.02 ^d^	3.00 ± 0.02 ^fg^	199.12
LNFS	16.26 ± 0.36 ^f^	1.32 ± 0.01 ^st^	0.47 ± 0.01 ^klmno^	2.37 ± 0.02 ^m^	50.01
FJSM05	25.66 ± 0.70 ^b^	1.34 ± 0.01 ^st^	0.44 ± 0.01 ^noppq^	1.82 ± 0.01 ^pq^	44.78
Mean	13.79	2.78	0.62	2.99	82.91
Standard deviation	5.36	2.04	0.43	1.58	46.57
CV/%	38.88	73.49	68.32	52.87	56.17

Data are presented as mean ± SD, and one-way ANOVA followed by Tukey’s HSD test was performed. Different lowercase letters in the same column indicate significant differences (*p* < 0.05).

**Table 2:** The contents of dietary fiber, resistant starch, pectin, kestose, and nystose in the 40 *Polygonatum* germplasm resources.

Sample Code	Dietary Fiber/% DW	Resistant Starch/% DW	Pectin/% DW	Kestose/% DW	Nystose/% DW
SCNC01	18.16 ± 0.02 ^p^	1.64 ± 0.03 ^cdefghi^	0.23 ± 0.01 ^fghi^	0.18 ± 0.00 ^v^	0.14 ± 0.00 ^no^
SCNC02	16.28 ± 0.11 ^u^	1.62 ± 0.03 ^defghij^	0.12 ± 0.00 ^mnop^	0.52 ± 0.00 ^h^	0.26 ± 0.04 ^efghijk^
JXYC	17.92 ± 0.09 ^q^	1.65 ± 0.04 ^cdefghi^	0.04 ± 0.03 ^q^	0.33 ± 0.00 ^lmn^	0.19 ± 0.01 ^jklmno^
GXGL	18.34 ± 0.04 ^p^	1.76 ± 0.04 ^b^	0.07 ± 0.03 ^pq^	0.72 ± 0.02 ^e^	0.40 ± 0.01 ^c^
GXHZ	23.33 ± 0.06 ^f^	1.59 ± 0.02 ^ghijkl^	0.10 ± 0.02 ^nop^	0.24 ± 0.02 ^pqrst^	0.15 ± 0.01 ^mno^
HNXH01	19.49 ± 0.10 ^l^	1.50 ± 0.01 ^no^	0.24 ± 0.01 ^efgh^	0.21 ± 0.01 ^tuv^	0.13 ± 0.01 ^no^
HNXH02	23.90 ± 0.11 ^d^	1.54 ± 0.02 ^jklmno^	0.08 ± 0.02 ^op^	0.25 ± 0.01 ^pqrst^	0.19 ± 0.01 ^jklmno^
HNXH03	20.08 ± 0.06 ^k^	1.65 ± 0.03 ^cdefghi^	0.14 ± 0.01 ^lmno^	0.37 ± 0.02 ^kl^	0.20 ± 0.01 ^ijklmn^
HNHH01	23.63 ± 0.04 ^e^	1.51 ± 0.02 ^mno^	0.25 ± 0.02 ^efg^	1.38 ± 0.07 ^a^	0.28 ± 0.01 ^efgh^
HNHH02	19.99 ± 0.10 ^k^	1.60 ± 0.03 ^efghijk^	0.12 ± 0.00 ^lmno^	1.08 ± 0.01 ^d^	0.28 ± 0.01 ^efg^
HNXX01	17.63 ± 0.10 ^r^	2.01 ± 0.03 ^a^	0.09 ± 0.01 ^op^	0.31 ± 0.01 ^mno^	0.23 ± 0.01 ^ghijklm^
HNXX02	21.39 ± 0.17 ^h^	1.71 ± 0.02 ^bc^	0.15 ± 0.03 ^klmn^	0.22 ± 0.01 ^rstuv^	0.17 ± 0.01 ^lmno^
AHLA	25.68 ± 0.07 ^a^	1.62 ± 0.04 ^defghi^	0.29 ± 0.01 ^e^	0.51 ± 0.01 ^h^	0.32 ± 0.02 ^de^
AHCZ01	18.57 ± 0.08 ^o^	1.67 ± 0.01 ^cdef^	0.18 ± 0.01 ^hijkl^	0.24 ± 0.00 ^pqrst^	0.17 ± 0.01 ^lmno^
AHCZ02	17.93 ± 0.05 ^q^	1.64 ± 0.02 ^cdefghi^	0.15 ± 0.02 ^klmn^	0.37 ± 0.03 ^jkl^	0.26 ± 0.01 ^efghijk^
AHCZ03	20.97 ± 0.08 ^i^	1.53 ± 0.01 ^klmno^	0.18 ± 0.02 ^hijkl^	0.51 ± 0.01 ^h^	0.23 ± 0.01 ^ghijklm^
AHCZ04	17.43 ± 0.13 ^rs^	1.51 ± 0.03 ^lmno^	0.12 ± 0.02 ^lmno^	0.60 ± 0.02 ^g^	0.36 ± 0.02 ^cd^
AHCZ05	17.89 ± 0.12 ^q^	1.57 ± 0.04 ^ijklmn^	0.08 ± 0.01 ^op^	0.38 ± 0.02 ^jkl^	0.27 ± 0.02 ^efghi^
ZJJN01	16.95 ± 0.11 ^t^	1.68 ± 0.02 ^cde^	0.42 ± 0.04 ^c^	0.22 ± 0.01 ^rstuv^	0.19 ± 0.00 ^klmno^
ZJJN02	18.86 ± 0.06 ^n^	1.59 ± 0.01 ^ghijkl^	0.18 ± 0.03 ^hijkl^	0.26 ± 0.01 ^opqrs^	0.20 ± 0.00 ^ijklmno^
ZJJN03	20.68 ± 0.05 ^j^	1.71 ± 0.01 ^bc^	0.24 ± 0.02 ^efg^	0.42 ± 0.01 ^ij^	0.32 ± 0.02 ^def^
ZJJN04	24.24 ± 0.12 ^c^	1.49 ± 0.03 ^o^	0.27 ± 0.01 ^ef^	0.29 ± 0.01 ^nop^	0.18 ± 0.00 ^lmno^
ZJJN05	17.45 ± 0.01 ^rs^	1.69 ± 0.02 ^bcd^	0.23 ± 0.00 ^fghi^	0.35 ± 0.01 ^klm^	0.18 ± 0.02 ^lmno^
ZJJS	14.89 ± 0.14 ^x^	1.61 ± 0.04 ^efghijk^	0.17 ± 0.02 ^ijklm^	0.34 ± 0.01 ^lm^	0.23 ± 0.01 ^ghijkl^
ZJAJ	15.86 ± 0.02 ^v^	1.03 ± 0.02 ^p^	0.42 ± 0.03 ^c^	0.27 ± 0.01 ^opq^	0.18 ± 0.00 ^lmno^
FJSM01	16.25 ± 0.14 ^u^	1.61 ± 0.01 ^efghijk^	0.16 ± 0.00 ^jklm^	0.51 ± 0.01 ^h^	0.31 ± 0.02 ^def^
FJSM02	17.33 ± 0.10 ^s^	1.03 ± 0.04 ^p^	0.51 ± 0.03 ^b^	0.46 ± 0.02 ^i^	0.28 ± 0.02 ^efg^
FJSM03	15.38 ± 0.01 ^w^	1.10 ± 0.05 ^p^	0.35 ± 0.03 ^d^	0.27 ± 0.00 ^opqr^	0.17 ± 0.01 ^lmno^
FJSM04	24.10 ± 0.01 ^cd^	1.08 ± 0.02 ^p^	0.65 ± 0.01 ^a^	1.33 ± 0.03 ^b^	0.23 ± 0.01 ^ghijklm^
FJNY	17.63 ± 0.09 ^r^	1.57 ± 0.02 ^ijklm^	0.20 ± 0.03 ^ghjik^	0.26 ± 0.01 ^pqrst^	0.14 ± 0.01 ^no^
FJNP	14.84 ± 0.05 ^x^	1.58 ± 0.02 ^hijklm^	0.09 ± 0.01 ^op^	0.27 ± 0.01 ^opq^	0.21 ± 0.01 ^hijklmn^
AHCZ06	25.35 ± 0.04 ^b^	1.50 ± 0.01 ^no^	0.29 ± 0.05 ^e^	0.19 ± 0.01 ^uv^	0.12 ± 0.01 ^o^
YNKM01	15.41 ± 0.05 ^w^	1.61 ± 0.03 ^defghij^	0.10 ± 0.01 ^nop^	0.66 ± 0.03 ^f^	0.54 ± 0.04 ^b^
YNKM02	19.25 ± 0.05 ^m^	1.61 ± 0.02 ^efghijk^	0.15 ± 0.02 ^klmn^	0.39 ± 0 ^jk^	0.25 ± 0.01 ^fghijkl^
YNKM03	14.88 ± 0.10 ^x^	1.67 ± 0.03 ^cdefg^	0.17 ± 0.01 ^jklm^	1.19 ± 0.03 ^c^	0.74 ± 0.13 ^a^
YNBS	25.82 ± 0.08 ^a^	1.66 ± 0.02 ^cdefgh^	0.22 ± 0.01 ^fghij^	0.25 ± 0.00 ^pqrst^	0.19 ± 0.01 ^jklmno^
HNHH03	23.67 ± 0.04 ^e^	1.47 ± 0.02 ^o^	0.36 ± 0.01 ^d^	1.21 ± 0.03 ^c^	0.31 ± 0.01 ^def^
SXLB	21.87 ± 0.03 ^g^	1.62 ± 0.03 ^defghi^	0.29 ± 0.01 ^e^	0.36 ± 0.01 ^kl^	0.27 ± 0.01 ^efghij^
LNFS	18.67 ± 0.04 ^no^	1.59 ± 0.02 ^fghijk^	0.27 ± 0.01 ^ef^	0.21 ± 0.01 ^stuv^	0.15 ± 0.01 ^mno^
FJSM05	19.45 ± 0.09 ^lm^	1.05 ± 0.02 ^p^	0.46 ± 0.03 ^bc^	0.23 ± 0.01 ^qrstu^	0.15 ± 0.01 ^mno^
Mean	19.44	1.55	0.22	0.46	0.25
Standard devision	3.24	0.21	0.13	0.33	0.12
CV/%	16.66	13.48	60.18	71.39	46.95

Data are presented as mean ± SD, and one-way ANOVA followed by Tukey’s HSD test was performed. Different lowercase letters in the same column indicate significant differences (*p* < 0.05).

**Table 3:** Other nutrient contents of 40 *Polygonatum* germplasm resources.

Sample Code	The Total Ash/% DW	Vitamin C/mg/100 g DW	Protein/% DW	Fe/mg/100 g DW	Ca/mg/100 g DW
SCNC01	2.23 ± 0.07 ^nop^	106.82 ± 0.97 ^hij^	4.59 ± 0.00 ^rs^	29.93 ± 0.09 ^f^	201.62 ± 2.21 ^op^
SCNC02	2.54 ± 0.07 ^jkl^	124.98 ± 1.56 ^e^	4.38 ± 0.00 ^st^	21.67 ± 0.15 ^lmn^	181.14 ± 5.50 ^qr^
JXYC	2.26 ± 0.05 ^nop^	98.79 ± 0.53 ^l^	5.25 ± 0.00 ^pq^	22.27 ± 0.16 ^lm^	194.56 ± 1.62 ^p^
GXGL	2.92 ± 0.05 ^ef^	99.02 ± 1.63 ^l^	5.91 ± 0.00 ^hij^	19.53 ± 0.09 ^o^	224.58 ± 3.82 ^m^
GXHZ	3.06 ± 0.05 ^de^	109.63 ± 0.90 ^gh^	6.82 ± 0.06 ^d^	31.19 ± 0.08 ^e^	269.42 ± 5.44 ^hi^
HNXH01	1.92 ± 0.03 ^q^	89.06 ± 1.23 ^o^	5.47 ± 0.00 ^mnopq^	12.59 ± 0.37 ^s^	192.8 ± 5.30 ^pq^
HNXH02	2.49 ± 0.07 ^jklm^	126.21 ± 2.00 ^e^	8.97 ± 0.00 ^a^	18.55 ± 0.68 ^op^	258.47 ± 2.12 ^ijk^
HNXH03	2.68 ± 0.09 ^ghij^	117.13 ± 1.94 ^f^	4.81 ± 0.00 ^r^	29.13 ± 0.39 ^f^	323.45 ± 0.61 ^e^
HNHH01	3.11 ± 0.09 ^cde^	166.53 ± 0.73 ^a^	6.13 ± 0.00 ^fgh^	44.4 ± 0.63 ^a^	288.84 ± 2.21 ^fg^
HNHH02	2.75 ± 0.07 ^fghi^	94.27 ± 0.73 ^mn^	5.83 ± 0.13 ^ijk^	34.42 ± 0.12 ^d^	220.69 ± 0.61 ^mn^
HNXX01	1.99 ± 0.09 ^q^	101.6 ± 1.07 ^kl^	5.83 ± 0.13 ^ijk^	25.54 ± 0.20 ^h^	280.37 ± 4.28 ^gh^
HNXX02	3.27 ± 0.02 ^c^	111.8 ± 2.44 ^g^	7.36 ± 0.13 ^c^	35.91 ± 0.67 ^c^	296.26 ± 2.21 ^f^
AHLA	2.83 ± 0.02 ^fg^	107.93 ± 0.81 ^ghij^	7.80 ± 0.13 ^b^	29.36 ± 0.50 ^f^	298.73 ± 2.80 ^f^
AHCZ01	2.42 ± 0.08 ^klmn^	105.00 ± 0.51 ^ijk^	5.54 ± 0.06 ^lmnop^	25.41 ± 0.27 ^hi^	210.45 ± 5.83 ^no^
AHCZ02	2.29 ± 0.07 ^mno^	136.12 ± 1.96 ^c^	5.80 ± 0.11 ^ijkl^	22.19 ± 0.64 ^lm^	289.19 ± 2.80 ^fg^
AHCZ03	3.11 ± 0.06 ^cde^	110.45 ± 0.41 ^gh^	5.25 ± 0.00 ^pq^	22.81 ± 0.66 ^kl^	253.53 ± 3.72 ^k^
AHCZ04	2.82 ± 0.08 ^fg^	131.43 ± 1.17 ^d^	6.31 ± 0.06 ^efg^	11.84 ± 0.71 st	267.3 ± 2.67 ^i^
AHCZ05	1.18 ± 0.07 ^s^	71.01 ± 0.98 ^rs^	3.06 ± 0.00 ^v^	9.98 ± 0.27 ^u^	210.1 ± 3.72 ^no^
ZJJN01	1.60 ± 0.09 ^r^	83.08 ± 1.03 ^p^	5.32 ± 0.13 ^opq^	11.25 ± 0.27 ^t^	199.15 ± 2.12 ^op^
ZJJN02	2.58 ± 0.08 ^ijkl^	67.79 ± 0.71 ^s^	5.58 ± 0.19 ^klmno^	16.80 ± 0.31 ^q^	312.50 ± 5.30 ^e^
ZJJN03	3.76 ± 0.07 ^a^	93.45 ± 0.51 ^n^	6.34 ± 0.00 ^ef^	12.75 ± 0.81 ^s^	438.91 ± 7.81 ^b^
ZJJN04	3.58 ± 0.03 ^b^	97.79 ± 1.30 ^lm^	6.56 ± 0.11 ^de^	25.10 ± 0.47 ^hi^	315.68 ± 6.62 ^e^
ZJJN05	2.69 ± 0.10 ^ghij^	109.75 ± 1.17 ^gh^	4.38 ± 0.00 ^st^	17.47 ± 0.29 ^pq^	281.07 ± 1.22 ^gh^
ZJJS	3.77 ± 0.04 ^a^	93.69 ± 1.27 ^mn^	5.32 ± 0.13 ^opq^	35.71 ± 0.39 ^c^	199.15 ± 2.8 ^op^
ZJAJ	2.53 ± 0.04 ^jkl^	86.13 ± 0.53 ^op^	4.12 ± 0.13 ^tu^	24.3 ± 0.34 ^ij^	180.79 ± 10.45 ^qr^
FJSM01	1.7 ± 0.08 ^r^	116.13 ± 0.57 ^f^	5.4 ± 0.13 ^nopq^	14.89 ± 0.19 ^r^	160.66 ± 1.62 ^s^
FJSM02	2.77 ± 0.09 ^de^	107.23 ± 1.40 ^ghij^	5.21 ± 0.13 ^q^	23.89 ± 0.36 ^jk^	190.68 ± 2.12 ^pq^
Mean	2.61	102.75	5.50	24.30	260.49
Standard devision	0.61	20.86	1.13	8.26	80.87
CV/%	23.31	20.30	20.46	34.00	31.05

Data are presented as mean ± SD, and one-way ANOVA followed by Tukey’s HSD test was performed. Different lowercase letters in the same column indicate significant differences (*p* < 0.05).

**Table 4:** Factor loadings, eigenvalues, and variance contribution rates of the 6 principal components.

Nutritional Components	PC1	PC2	PC3	PC4	PC5	PC6
Polysaccharide	−0.078	−0.490	0.068	0.116	0.329	0.036
The total ash	0.352	−0.088	0.027	−0.088	0.071	0.666
Vitamin C	0.258	0.098	0.28	0.359	0.003	−0.272
Resistant starch	−0.073	0.478	0.291	−0.242	−0.130	−0.056
Protein	0.18	−0.021	0.497	−0.032	0.492	0.177
Fructose	0.378	0.058	0.085	−0.159	−0.147	−0.126
Glucose	0.384	−0.089	−0.246	−0.122	−0.106	−0.283
Sucrose	0.29	0.274	0.02	0.455	0.127	−0.119
Kestose	0.31	0.223	−0.395	0.22	0.198	−0.076
Nistose trihydrate	0.038	0.369	−0.402	−0.040	0.378	0.309
Pectin	0.164	−0.455	−0.286	−0.025	0.118	−0.201
Fe	0.224	−0.160	0.097	0.412	−0.564	0.382
Ca	0.317	0.001	−0.100	−0.517	−0.197	0.074
TDF	0.341	−0.100	0.312	−0.235	0.161	−0.209
Eigenvalue	4.26	2.61	2.01	1.24	0.91	0.71
Variance explained (%)	30.43	18.61	14.33	8.88	6.47	5.1
Cumulative variance explained (%)	30.43	49.04	63.38	72.26	78.72	83.83

**Table 5:** Principal component scores, nutritional quality comprehensive scores, and ranking of the 40 *Polygonatum* germplasm resources.

Sample Code	Y1	Y2	Y3	Y4	Y5	Y6	Y Score	Rank
HNHH01	2.696	0.945	0.055	2.686	−0.566	−0.973	1.156	1
HNHH03	1.733	0.772	−0.549	1.117	1.547	−1.047	0.738	2
HNHH02	0.891	1.28	−0.357	1.92	−0.054	0.284	0.64	3
GXHZ	0.83	−0.015	1.684	−0.062	−0.357	0.481	0.487	4
HNXX02	0.506	0.101	1.536	−0.237	−0.752	1.511	0.4	5
AHLA	1.062	−0.258	0.729	−0.770	1.112	0.095	0.388	6
HNXH02	−0.097	−0.770	2.23	−0.123	2.796	0.105	0.322	7
YNBS	1.147	−0.213	1.241	−0.939	−1.174	−0.161	0.32	8
AHCZ04	−0.047	1.054	0.039	0.166	1.398	0.149	0.3	9
ZJJN04	0.766	−0.574	0.804	−0.826	0.381	0.91	0.239	10
ZJJN03	0.788	0.541	0	−2.399	0.664	1.147	0.229	11
GXGL	−0.378	1.26	−0.130	−0.068	0.997	1.308	0.226	12
AHCZ02	0.015	0.659	0.532	0.565	0.05	−0.648	0.224	13
AHCZ03	0.545	−0.084	0.218	0.156	0.271	−0.040	0.211	14
SXLB	0.848	0.164	0.393	−0.908	−0.061	−1.082	0.205	15
HNXH03	0.192	0.405	0.367	−0.047	−1.292	0.002	0.099	16
ZJJS	−0.038	0.032	−0.019	0.508	−1.302	2.58	0.084	17
HNXX01	−0.358	1.131	0.764	−0.518	−1.009	−0.480	0.075	18
SCNC02	−0.431	0.74	−0.179	0.959	−0.436	−0.336	0.021	19
YNKM03	−0.295	1.897	−2.730	−0.210	1.458	1.369	0.017	20
AHCZ06	−0.030	−0.628	1.057	−0.804	0.539	−0.189	−0.021	21
FJSM04	3.161	−1.959	−2.586	−1.807	−0.828	−1.126	−0.045	22
FJSM01	−0.845	0.782	−0.210	0.526	0.627	−1.296	−0.121	23
AHCZ01	−0.657	−0.389	0.686	0.366	0.04	0.058	−0.136	24
JXYC	−0.944	0.028	0.536	0.474	0.197	0.108	−0.145	25
YNKM01	−0.571	1.397	−1.699	−0.752	0.147	1.302	−0.148	26
ZJJN05	−0.403	0.279	−0.099	−0.257	−0.526	−0.452	−0.165	27
FJNY	−0.669	0.168	0.456	0.449	−1.034	−0.988	−0.184	28
FJNP	−0.815	0.282	0.045	0.796	−1.978	−0.185	−0.256	29
ZJJN02	−0.578	0.114	−0.073	−1.534	−0.209	0.414	−0.294	30
SCNC01	−0.782	−0.283	0.344	0.413	−1.347	−0.279	−0.306	31
FJSM02	−0.238	−1.606	−1.091	1.29	1.285	0.257	−0.317	32
FJSM03	−0.528	−1.882	−0.449	1.316	−0.114	1.949	−0.366	33
LNFS	−0.959	−0.463	0.107	−0.086	0.204	−0.810	−0.399	34
HNXH01	−0.988	−0.514	0.287	−0.346	0.679	−1.346	−0.411	35
YNKM02	−0.642	0.278	−0.692	−1.265	−1.549	0.425	−0.434	36
FJSM05	−0.339	−2.469	−0.679	0.832	−0.488	0.568	−0.589	37
ZJJN01	−1.292	−0.763	−0.268	−0.618	1.008	−1.652	−0.647	38
AHCZ05	−1.428	0.724	−1.131	−0.828	−0.625	−2.057	−0.681	39
ZJAJ	−0.826	−2.162	−1.165	0.866	0.3	0.124	−0.718	40

## Data Availability

The original contributions presented in the study are included in the article/supplementary material, further inquiries can be directed to the corresponding author.

## Supplementary Materials

The following supporting information can be downloaded at https://www.mdpi.com/article/10.3390/foods14213663/s1: Table S1: Plant material information; Table S2: Correlation analysis of nutritional quality indicators of the 40 *Polygonatum* germplasm resources; Table S3: Membership function analysis of nutritional components in the 40 *Polygonatum* germplasm resources.
