# Supplementary material for: Comparison of Major Nutritional Components in Polygonatum Germplasm Resources from Different Origins
Source: Foods. 2025 Oct 27;14(21):3663. doi: 10.3390/foods14213663 (PMC12609961; doi:10.3390/foods14213663)
Supplement: Supplementary file 1 [file foods-14-03663-s001.zip › foods-3919034-supplementary.pdf]

Table S1. Plant Material information

No.	Sample code	Subspecies	Origin
1	SCNC01	*Polygonatum cyrtonema* Hua	Sichuan
2	SCNC02	*Polygonatum cyrtonema* Hua	Sichuan
3	JXYC	*Polygonatum cyrtonema* Hua	Jiangxi
4	GXGL	*Polygonatum cyrtonema* Hua	Guangxi
5	GXHZ	*Polygonatum cyrtonema* Hua	Guangxi
6	HNXH01	*Polygonatum cyrtonema* Hua	Hunan
7	HNXH02	*Polygonatum cyrtonema* Hua	Hunan
8	HNXH03	*Polygonatum cyrtonema* Hua	Hunan
9	HNHH01	*Polygonatum cyrtonema* Hua	Hunan
10	HNHH02	*Polygonatum cyrtonema* Hua	Hunan
11	HNXX01	*Polygonatum cyrtonema* Hua	Hunan
12	HNXX02	*Polygonatum cyrtonema* Hua	Hunan
13	AHLA	*Polygonatum cyrtonema* Hua	Anhui
14	AHCZ01	*Polygonatum cyrtonema* Hua	Anhui
15	AHCZ02	*Polygonatum cyrtonema* Hua	Anhui
16	AHCZ03	*Polygonatum cyrtonema* Hua	Anhui

No.	Sample code	Subspecies	Origin
17	AHCZ04	*Polygonatum cyrtonema* Hua	Anhui
18	AHCZ05	*Polygonatum cyrtonema* Hua	Anhui
19	ZJJN01	*Polygonatum cyrtonema* Hua	Zhejiang
20	ZJJN02	*Polygonatum cyrtonema* Hua	Zhejiang
21	ZJJN03	*Polygonatum cyrtonema* Hua	Zhejiang
22	ZJJN04	*Polygonatum cyrtonema* Hua	Zhejiang
23	ZJJN05	*Polygonatum cyrtonema* Hua	Zhejiang
24	ZJJS	*Polygonatum cyrtonema* Hua	Zhejiang
25	ZJAJ	*Polygonatum cyrtonema* Hua	Zhejiang
26	FJSM01	*Polygonatum cyrtonema* Hua	Fujian
27	FJSM02	*Polygonatum cyrtonema* Hua	Fujian
28	FJSM03	*Polygonatum cyrtonema* Hua	Fujian
29	FJSM04	*Polygonatum cyrtonema* Hua	Fujian
30	FJNY	*Polygonatum cyrtonema* Hua	Fujian
31	FJNP	*Polygonatum cyrtonema* Hua	Fujian
32	AHCZ06	*Polygonatum odoratum* (Mill.) Druce	Anhui
33	YNKM01	*Polygonatum kingianum* Coll. et Hemsl.	Yunnan
34	YNKM02	*Polygonatum kingianum* Coll. et Hemsl.	Yunnan

No.	Sample code	Subspecies	Origin
35	YNKM03	*Polygonatum kingianum* Coll. et Hemsl.	Yunnan
36	YNBS	*Polygonatum kingianum* Coll. et Hemsl.	Yunnan
37	HNHH03	*Polygonatum sibiricum* Red.	Hunan
38	SXLB	*Polygonatum sibiricum* Red.	Shanxi
39	LNFS	*Polygonatum sibiricum* Red.	Liaoning
40	FJSM05	*Polygonatum filipes* Merr. ex C. Jeffrey & McEwan	Fujian

Table S2. Correlation analysis of nutritional quality indicators of 40 *Polygonatum* germplasm resources

Nutritional components	Poly-saccharide	The total ash	Vitamin C	Resistant starch	Protein	Fructose	Glucose	Sucrose	Kestose	Nistose trihydrate	Pectin	Fe	Ca	TDF
Poly-saccharide	1.000													
The total ash	−0.015	1.000												
VitaminC	−0.069	0.245	1.000											
Resistant starch	−0.548**	−0.213	0.098	1.000										
Protein	0.113	0.364*	0.379*	0.159	1.000									
Fructose	−0.215	0.520**	0.385*	0.045	0.249	1.000								
Glucose	−0.091	0.436**	0.240	−0.269	0.054	0.575**	1.000							
Sucrose	−0.363*	0.309	0.478**	0.108	0.212	0.427**	0.279	1.000						
Kestose	−0.327*	0.323*	0.264	−0.112	−0.080	0.331*	0.650**	0.668**	1.000					

Nutritional components	Poly-saccharide	The total ash	Vitamin C	Resistant starch	Protein	Fructose	Glucose	Sucrose	Kestose	Nistose trihydrate	Pectin	Fe	Ca	TDF
Nistose trihydrate	−0.362*	0.056	−0.089	0.174	−0.185	0.031	0.073	0.205	0.605**	1.000				
Pectin	0.418**	0.278	−0.078	−0.741**	−0.079	0.153	0.509**	−0.095	0.172	−0.191	1.000			
Fe	0.073	0.382*	0.322*	−0.250	0.110	0.277	0.306	0.265	0.146	−0.265	0.158	1.000		
Ca	−0.176	0.512**	0.167	0.012	0.066	0.494**	0.608**	0.066	0.338*	0.125	0.222	0.145	1.000	
TDF	0.046	0.453**	0.359*	−0.012	0.595**	0.586**	0.445**	0.313*	0.144	−0.262	0.212	0.216	0.493**	1.000

Note: * indicates a significant correlation ($p < 0.05$), and ** indicates a highly significant correlation ($p < 0.01$).

Table S3. Membership function analysis of nutritional components in 40 *Polygonatum* germplasm resources

Sample code	Poly-saccharide	The total ash	Vitamin C	Resistant starch	Protein	Fructose	Glucose	Sucrose	Kestose	Nistose trihydrate	Pectin	Fe	Ca	TDF	Average Member-ship Value	Rank
HNHH01	0.05	0.74	1	0.49	0.52	0.63	0.45	1	1	0.25	0.34	1	0.32	0.8	0.61	1
FJSM04	0.44	1	0.42	0.05	0.29	0.68	1	0.1	0.96	0.17	1	0.63	1	0.84	0.61	2
AHLA	0.37	0.63	0.41	0.6	0.8	0.32	0.35	0.17	0.27	0.32	0.41	0.56	0.34	0.99	0.47	4
GXHZ	0.39	0.72	0.42	0.57	0.64	0.81	0.04	0.42	0.05	0.05	0.09	0.62	0.27	0.77	0.42	6
HNHH02	0	0.6	0.27	0.58	0.47	0.21	0.22	0.99	0.75	0.26	0.13	0.71	0.15	0.47	0.41	7
ZJJN04	0.33	0.91	0.3	0.47	0.59	0.36	0.12	0.29	0.09	0.09	0.37	0.44	0.38	0.86	0.4	9
ZJJN03	0.1	0.98	0.26	0.7	0.56	0.43	0.17	0.23	0.2	0.31	0.32	0.08	0.69	0.53	0.4	10
AHCZ03	0.53	0.74	0.43	0.51	0.37	0.52	0.17	0.4	0.28	0.17	0.23	0.37	0.23	0.56	0.39	11
HNXH02	1	0.5	0.59	0.52	1	0.04	0.02	0.09	0.06	0.11	0.06	0.25	0.24	0.82	0.38	12
HNXX02	0.1	0.8	0.45	0.69	0.73	0.26	0.09	0.19	0.03	0.08	0.18	0.75	0.34	0.6	0.38	13
HNXH03	0.15	0.57	0.5	0.63	0.3	0.25	0.09	0.3	0.16	0.13	0.16	0.56	0.4	0.48	0.33	14
AHCZ02	0.24	0.42	0.69	0.62	0.46	0.22	0.06	0.41	0.16	0.23	0.18	0.35	0.32	0.28	0.33	15
ZJJS	0.17	0.99	0.26	0.59	0.38	0.28	0.12	0.14	0.13	0.18	0.21	0.75	0.1	0	0.31	17

Sample code	Poly-saccharide	The total ash	Vitamin C	Resistant starch	Protein	Fructose	Glucose	Sucrose	Kestose	Nistose trihydrate	Pectin	Fe	Ca	TDF	Average Member-ship Value	Rank
HNXX01	0.12	0.31	0.34	1	0.47	0.29	0.12	0.25	0.11	0.17	0.07	0.45	0.3	0.25	0.3	19
GXGL	0.12	0.66	0.32	0.75	0.48	0	0	0.19	0.45	0.45	0.04	0.28	0.16	0.32	0.3	20
FJSM02	0.61	0.6	0.4	0	0.36	0.03	0.04	0.2	0.23	0.26	0.77	0.4	0.07	0.23	0.3	22
AHCZ04	0.03	0.63	0.64	0.49	0.55	0.13	0.01	0.3	0.35	0.38	0.13	0.05	0.26	0.24	0.3	23
AHCZ01	0.64	0.47	0.38	0.66	0.42	0.06	0.02	0.15	0.05	0.08	0.23	0.45	0.12	0.34	0.29	24
ZJJN05	0.21	0.58	0.42	0.67	0.22	0.11	0.05	0.25	0.14	0.1	0.31	0.22	0.3	0.24	0.27	25
FJSM03	0.71	0.71	0.14	0.07	0.44	0.04	0.05	0.07	0.07	0.08	0.5	0.77	0.04	0.05	0.27	26
SCNC02	0.16	0.52	0.58	0.6	0.22	0.16	0.08	0.26	0.28	0.23	0.12	0.34	0.05	0.13	0.27	27
JXYC	0.63	0.41	0.31	0.63	0.37	0.01	0	0.18	0.12	0.11	0	0.36	0.08	0.28	0.25	29
SCNC01	0.26	0.4	0.4	0.63	0.26	0.01	0.03	0.04	0	0.03	0.31	0.58	0.1	0.3	0.24	30
ZJAJ	0.88	0.52	0.19	0	0.18	0.08	0.07	0.01	0.08	0.09	0.63	0.42	0.05	0.09	0.23	31
ZJJN02	0.09	0.53	0	0.57	0.43	0.15	0.05	0.07	0.07	0.12	0.22	0.2	0.38	0.37	0.23	33
FJSM01	0.14	0.2	0.49	0.59	0.4	0.13	0.06	0.14	0.28	0.31	0.19	0.14	0	0.13	0.23	34
ZJJN01	0.64	0.16	0.15	0.66	0.38	0.01	0.04	0	0.03	0.11	0.62	0.04	0.1	0.19	0.22	37
HNXH01	0.44	0.28	0.22	0.48	0.41	0.07	0.03	0.1	0.02	0.02	0.32	0.08	0.08	0.42	0.21	39
AHCZ05	0.08	0	0.03	0.55	0	0.09	0.12	0.08	0.16	0.24	0.06	0	0.12	0.28	0.13	40
